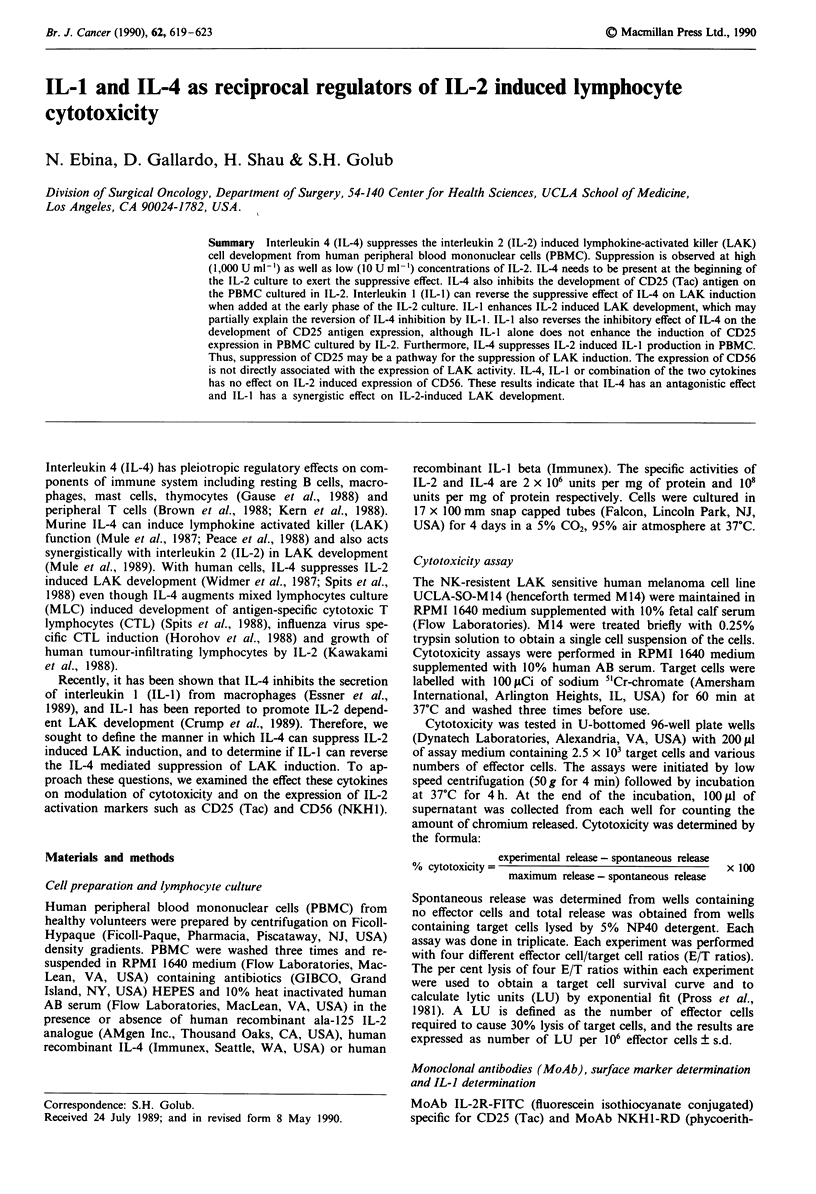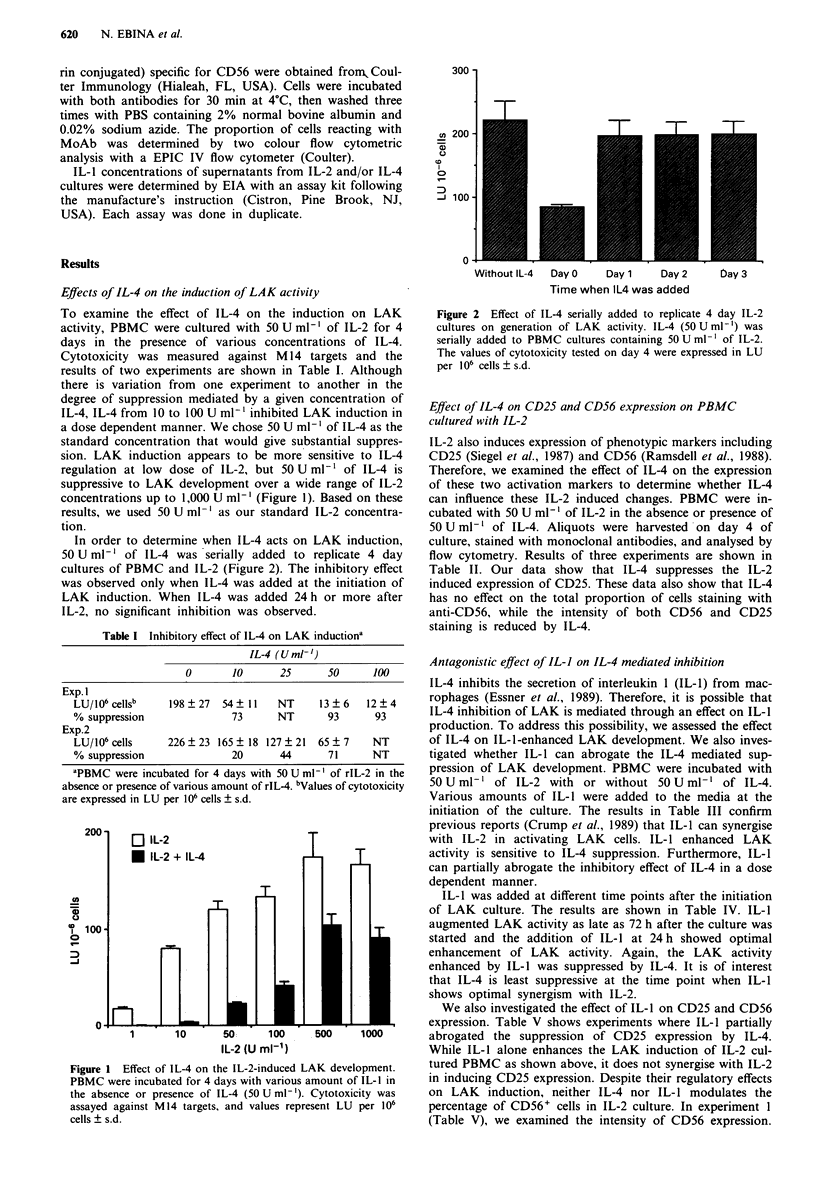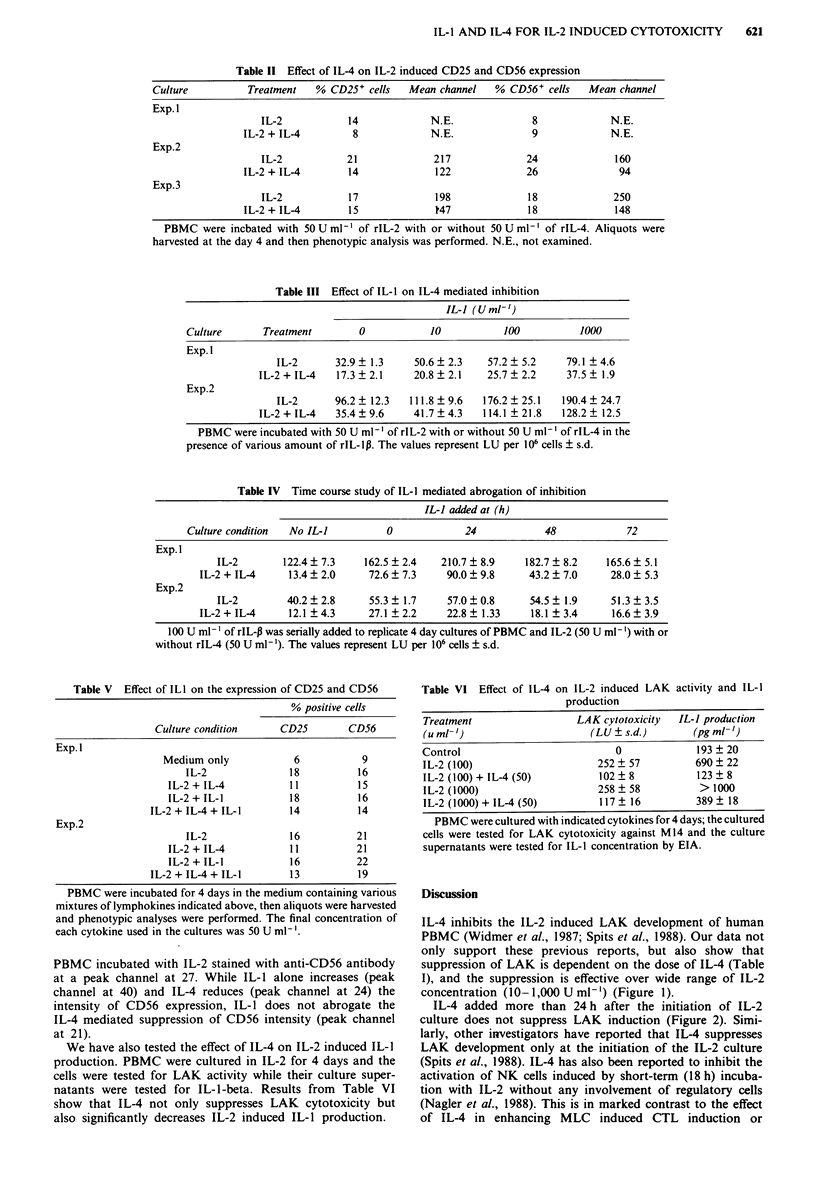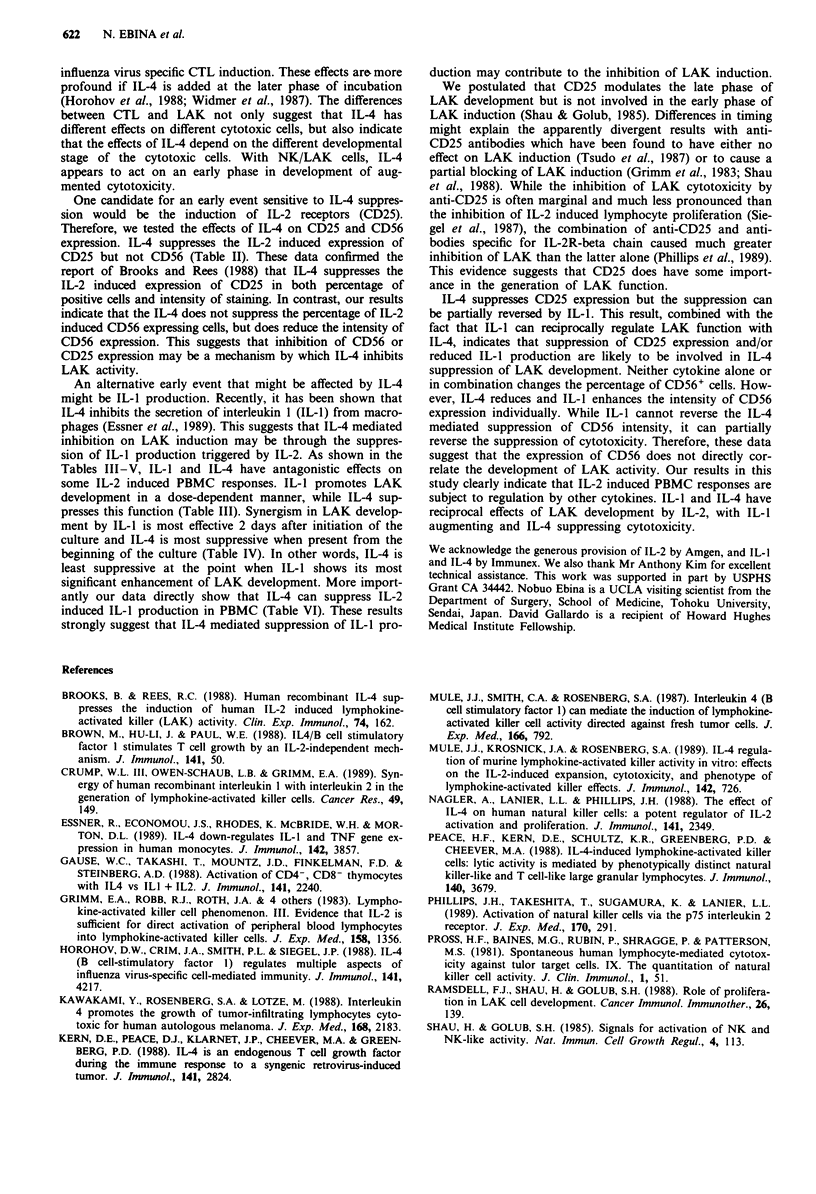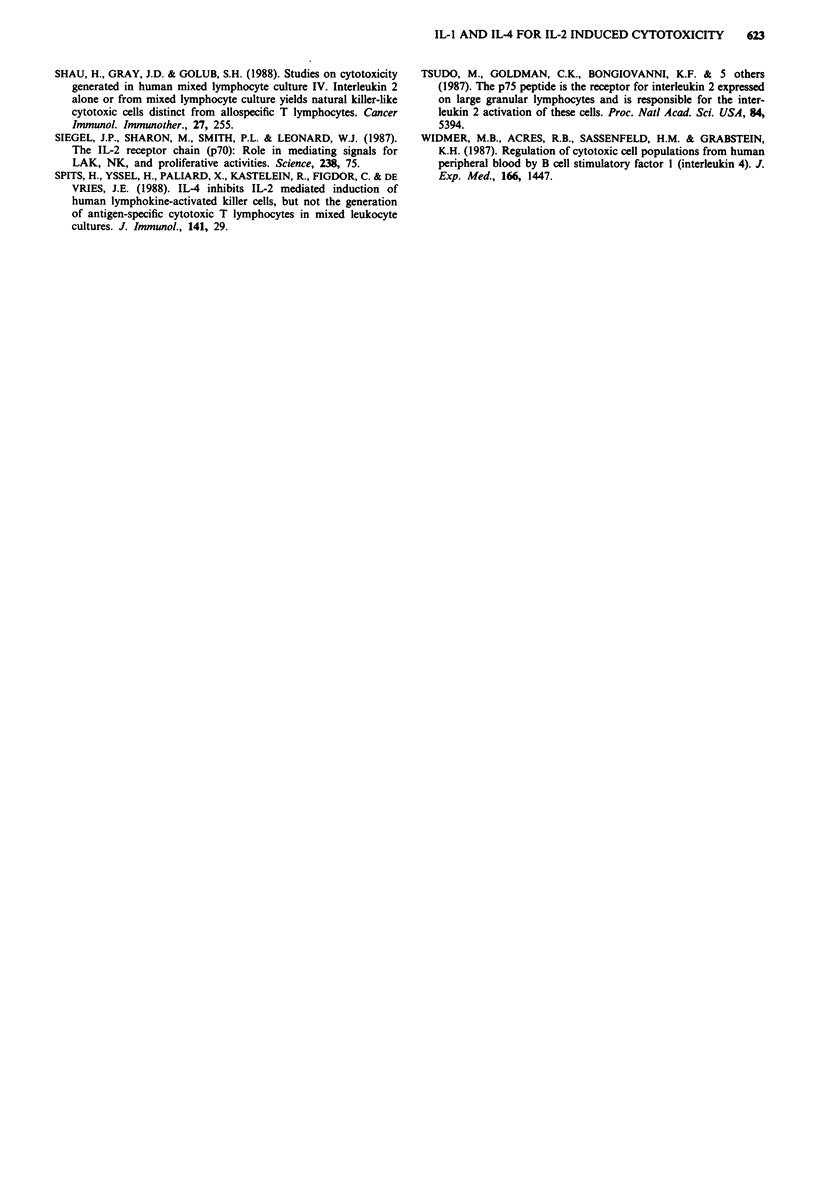# IL-1 and IL-4 as reciprocal regulators of IL-2 induced lymphocyte cytotoxicity.

**DOI:** 10.1038/bjc.1990.341

**Published:** 1990-10

**Authors:** N. Ebina, D. Gallardo, H. Shau, S. H. Golub

**Affiliations:** Department of Surgery, UCLA School of Medicine 90024-1782.

## Abstract

Interleukin 4 (IL-4) suppresses the interleukin 2 (IL-2) induced lymphokine-activated killer (LAK) cell development from human peripheral blood mononuclear cells (PBMC). Suppression is observed at high (1,000 U ml-1) as well as low (10 U ml-1) concentrations of IL-2. IL-4 needs to be present at the beginning of the IL-2 culture to exert the suppressive effect. IL-4 also inhibits the development of CD25 (Tac) antigen on the PBMC cultured in IL-2. Interleukin 1 (IL-1) can reverse the suppressive effect of IL-4 on LAK induction when added at the early phase of the IL-2 culture. IL-1 enhances IL-2 induced LAK development, which may partially explain the reversion of IL-4 inhibition by IL-1. IL-1 also reverses the inhibitory effect of IL-4 on the development of CD25 antigen expression, although IL-1 alone does not enhance the induction of CD25 expression in PBMC cultured by IL-2. Furthermore, IL-4 suppresses IL-2 induced IL-1 production in PBMC. Thus, suppression of CD25 may be a pathway for the suppression of LAK induction. The expression of CD56 is not directly associated with the expression of LAK activity. IL-4, IL-1 or combination of the two cytokines has no effect on IL-2 induced expression of CD56. These results indicate that IL-4 has an antagonistic effect and IL-1 has a synergistic effect on IL-2-induced LAK development.


Br. J. Cancer (1990), 62, 619-623 © Macmillan Press Ltd., 1990

### IL-1 and IL-4 as reciprocal regulators of IL-2 induced lymphocyte cytotoxicity

N. Ebina, D. Gallardo, H. Shau & S.H. Golub

*Division of Surgical Oncology, Department of Surgery, 54-140 Center for Health Sciences, UCLA School of Medicine, Los Angeles, CA 90024-1782, USA.*

**Summary** Interleukin 4 (IL-4) suppresses the interleukin 2 (IL-2) induced lymphokine-activated killer (LAK) cell development from human peripheral blood mononuclear cells (PBMC). Suppression is observed at high (1,000 U $ml^{-1}$) as well as low (10 U $ml^{-1}$) concentrations of IL-2. IL-4 needs to be present at the beginning of the IL-2 culture to exert the suppressive effect. IL-4 also inhibits the development of CD25 (Tac) antigen on the PBMC cultured in IL-2. Interleukin 1 (IL-1) can reverse the suppressive effect of IL-4 on LAK induction when added at the early phase of the IL-2 culture. IL-1 enhances IL-2 induced LAK development, which may partially explain the reversion of IL-4 inhibition by IL-1. IL-1 also reverses the inhibitory effect of IL-4 on the development of CD25 antigen expression, although IL-1 alone does not enhance the induction of CD25 expression in PBMC cultured by IL-2. Furthermore, IL-4 suppresses IL-2 induced IL-1 production in PBMC. Thus, suppression of CD25 may be a pathway for the suppression of LAK induction. The expression of CD56 is not directly associated with the expression of LAK activity. IL-4, IL-1 or combination of the two cytokines has no effect on IL-2 induced expression of CD56. These results indicate that IL-4 has an antagonistic effect and IL-1 has a synergistic effect on IL-2-induced LAK development.

Interleukin 4 (IL-4) has pleiotropic regulatory effects on components of immune system including resting B cells, macrophages, mast cells, thymocytes (Gause *et al.*, 1988) and peripheral T cells (Brown *et al.*, 1988; Kern *et al.*, 1988). Murine IL-4 can induce lymphokine activated killer (LAK) function (Mule *et al.*, 1987; Peace *et al.*, 1988) and also acts synergistically with interleukin 2 (IL-2) in LAK development (Mule *et al.*, 1989). With human cells, IL-4 suppresses IL-2 induced LAK development (Widmer *et al.*, 1987; Spits *et al.*, 1988) even though IL-4 augments mixed lymphocytes culture (MLC) induced development of antigen-specific cytotoxic T lymphocytes (CTL) (Spits *et al.*, 1988), influenza virus specific CTL induction (Horohov *et al.*, 1988) and growth of human tumour-infiltrating lymphocytes by IL-2 (Kawakami *et al.*, 1988).

Recently, it has been shown that IL-4 inhibits the secretion of interleukin 1 (IL-1) from macrophages (Essner *et al.*, 1989), and IL-1 has been reported to promote IL-2 dependent LAK development (Crump *et al.*, 1989). Therefore, we sought to define the manner in which IL-4 can suppress IL-2 induced LAK induction, and to determine if IL-1 can reverse the IL-4 mediated suppression of LAK induction. To approach these questions, we examined the effect these cytokines on modulation of cytotoxicity and on the expression of IL-2 activation markers such as CD25 (Tac) and CD56 (NKH1).

### Materials and methods

### *Cell preparation and lymphocyte culture*

Human peripheral blood mononuclear cells (PBMC) from healthy volunteers were prepared by centrifugation on Ficoll-Hypaque (Ficoll-Paque, Pharmacia, Piscataway, NJ, USA) density gradients. PBMC were washed three times and resuspended in RPMI 1640 medium (Flow Laboratories, MacLean, VA, USA) containing antibiotics (GIBCO, Grand Island, NY, USA) HEPES and 10% heat inactivated human AB serum (Flow Laboratories, MacLean, VA, USA) in the presence or absence of human recombinant ala-125 IL-2 analogue (AMgen Inc., Thousand Oaks, CA, USA), human recombinant IL-4 (Immunex, Seattle, WA, USA) or human recombinant IL-1 beta (Immunex). The specific activities of IL-2 and IL-4 are $2 \times 10^6$ units per mg of protein and $10^8$ units per mg of protein respectively. Cells were cultured in 17 × 100 mm snap capped tubes (Falcon, Lincoln Park, NJ, USA) for 4 days in a 5% $CO_2$, 95% air atmosphere at 37°C.

### *Cytotoxicity assay*

The NK-resistent LAK sensitive human melanoma cell line UCLA-SO-M14 (henceforth termed M14) were maintained in RPMI 1640 medium supplemented with 10% fetal calf serum (Flow Laboratories). M14 were treated briefly with 0.25% trypsin solution to obtain a single cell suspension of the cells. Cytotoxicity assays were performed in RPMI 1640 medium supplemented with 10% human AB serum. Target cells were labelled with 100 μCi of sodium $^{51}$Cr-chromate (Amersham International, Arlington Heights, IL, USA) for 60 min at 37°C and washed three times before use.

Cytotoxicity was tested in U-bottomed 96-well plate wells (Dynatech Laboratories, Alexandria, VA, USA) with 200 μl of assay medium containing $2.5 \times 10^3$ target cells and various numbers of effector cells. The assays were initiated by low speed centrifugation (50 *g* for 4 min) followed by incubation at 37°C for 4 h. At the end of the incubation, 100 μl of supernatant was collected from each well for counting the amount of chromium released. Cytotoxicity was determined by the formula:

$$\% \text{ cytotoxicity} = \frac{\text{experimental release} - \text{spontaneous release}}{\text{maximum release} - \text{spontaneous release}} \times 100$$

Spontaneous release was determined from wells containing no effector cells and total release was obtained from wells containing target cells lysed by 5% NP40 detergent. Each assay was done in triplicate. Each experiment was performed with four different effector cell/target cell ratios (E/T ratios). The per cent lysis of four E/T ratios within each experiment were used to obtain a target cell survival curve and to calculate lytic units (LU) by exponential fit (Pross *et al.*, 1981). A LU is defined as the number of effector cells required to cause 30% lysis of target cells, and the results are expressed as number of LU per $10^6$ effector cells ± s.d.

### *Monoclonal antibodies (MoAb), surface marker determination and IL-1 determination*

MoAb IL-2R-FITC (fluorescein isothiocyanate conjugated) specific for CD25 (Tac) and MoAb NKH1-RD (phycoerith-

Correspondence: S.H. Golub.
Received 24 July 1989; and in revised form 8 May 1990.

rin conjugated) specific for CD56 were obtained from Coulter Immunology (Hialeah, FL, USA). Cells were incubated with both antibodies for 30 min at 4°C, then washed three times with PBS containing 2% normal bovine albumin and 0.02% sodium azide. The proportion of cells reacting with MoAb was determined by two colour flow cytometric analysis with a EPIC IV flow cytometer (Coulter).

IL-1 concentrations of supernatants from IL-2 and/or IL-4 cultures were determined by EIA with an assay kit following the manufacture's instruction (Cistron, Pine Brook, NJ, USA). Each assay was done in duplicate.

### Results

### *Effects of IL-4 on the induction of LAK activity*

To examine the effect of IL-4 on the induction on LAK activity, PBMC were cultured with 50 U ml$^{-1}$ of IL-2 for 4 days in the presence of various concentrations of IL-4. Cytotoxicity was measured against M14 targets and the results of two experiments are shown in Table I. Although there is variation from one experiment to another in the degree of suppression mediated by a given concentration of IL-4, IL-4 from 10 to 100 U ml$^{-1}$ inhibited LAK induction in a dose dependent manner. We chose 50 U ml$^{-1}$ of IL-4 as the standard concentration that would give substantial suppression. LAK induction appears to be more sensitive to IL-4 regulation at low dose of IL-2, but 50 U ml$^{-1}$ of IL-4 is suppressive to LAK development over a wide range of IL-2 concentrations up to 1,000 U ml$^{-1}$ (Figure 1). Based on these results, we used 50 U ml$^{-1}$ as our standard IL-2 concentration.

In order to determine when IL-4 acts on LAK induction, 50 U ml$^{-1}$ of IL-4 was serially added to replicate 4 day cultures of PBMC and IL-2 (Figure 2). The inhibitory effect was observed only when IL-4 was added at the initiation of LAK induction. When IL-4 was added 24 h or more after IL-2, no significant inhibition was observed.

**Table I** Inhibitory effect of IL-4 on LAK induction[a]

| | *IL-4 (U ml$^{-1}$)* | | | | |
|---|---|---|---|---|---|
| | *0* | *10* | *25* | *50* | *100* |
| Exp.1 | | | | | |
| LU/10$^6$ cells[b] | 198 ± 27 | 54 ± 11 | NT | 13 ± 6 | 12 ± 4 |
| % suppression | | 73 | NT | 93 | 93 |
| Exp.2 | | | | | |
| LU/10$^6$ cells | 226 ± 23 | 165 ± 18 | 127 ± 21 | 65 ± 7 | NT |
| % suppression | | 20 | 44 | 71 | NT |

[a]PBMC were incubated for 4 days with 50 U ml$^{-1}$ of rIL-2 in the absence or presence of various amount of rIL-4. [b]Values of cytotoxicity are expressed in LU per 10$^6$ cells ± s.d.

**Figure 1** Effect of IL-4 on the IL-2-induced LAK development. PBMC were incubated for 4 days with various amount of IL-1 in the absence or presence of IL-4 (50 U ml$^{-1}$). Cytotoxicity was assayed against M14 targets, and values represent LU per 10$^6$ cells ± s.d.

**Figure 2** Effect of IL-4 serially added to replicate 4 day IL-2 cultures on generation of LAK activity. IL-4 (50 U ml$^{-1}$) was serially added to PBMC cultures containing 50 U ml$^{-1}$ of IL-2. The values of cytotoxicity tested on day 4 were expressed in LU per 10$^6$ cells ± s.d.

### *Effect of IL-4 on CD25 and CD56 expression on PBMC cultured with IL-2*

IL-2 also induces expression of phenotypic markers including CD25 (Siegel *et al.*, 1987) and CD56 (Ramsdell *et al.*, 1988). Therefore, we examined the effect of IL-4 on the expression of these two activation markers to determine whether IL-4 can influence these IL-2 induced changes. PBMC were incubated with 50 U ml$^{-1}$ of IL-2 in the absence or presence of 50 U ml$^{-1}$ of IL-4. Aliquots were harvested on day 4 of culture, stained with monoclonal antibodies, and analysed by flow cytometry. Results of three experiments are shown in Table II. Our data show that IL-4 suppresses the IL-2 induced expression of CD25. These data also show that IL-4 has no effect on the total proportion of cells staining with anti-CD56, while the intensity of both CD56 and CD25 staining is reduced by IL-4.

### *Antagonistic effect of IL-1 on IL-4 mediated inhibition*

IL-4 inhibits the secretion of interleukin 1 (IL-1) from macrophages (Essner *et al.*, 1989). Therefore, it is possible that IL-4 inhibition of LAK is mediated through an effect on IL-1 production. To address this possibility, we assessed the effect of IL-4 on IL-1-enhanced LAK development. We also investigated whether IL-1 can abrogate the IL-4 mediated suppression of LAK development. PBMC were incubated with 50 U ml$^{-1}$ of IL-2 with or without 50 U ml$^{-1}$ of IL-4. Various amounts of IL-1 were added to the media at the initiation of the culture. The results in Table III confirm previous reports (Crump *et al.*, 1989) that IL-1 can synergise with IL-2 in activating LAK cells. IL-1 enhanced LAK activity is sensitive to IL-4 suppression. Furthermore, IL-1 can partially abrogate the inhibitory effect of IL-4 in a dose dependent manner.

IL-1 was added at different time points after the initiation of LAK culture. The results are shown in Table IV. IL-1 augmented LAK activity as late as 72 h after the culture was started and the addition of IL-1 at 24 h showed optimal enhancement of LAK activity. Again, the LAK activity enhanced by IL-1 was suppressed by IL-4. It is of interest that IL-4 is least suppressive at the time point when IL-1 shows optimal synergism with IL-2.

We also investigated the effect of IL-1 on CD25 and CD56 expression. Table V shows experiments where IL-1 partially abrogated the suppression of CD25 expression by IL-4. While IL-1 alone enhances the LAK induction of IL-2 cultured PBMC as shown above, it does not synergise with IL-2 in inducing CD25 expression. Despite their regulatory effects on LAK induction, neither IL-4 nor IL-1 modulates the percentage of CD56$^+$ cells in IL-2 culture. In experiment 1 (Table V), we examined the intensity of CD56 expression.

**Table II** Effect of IL-4 on IL-2 induced CD25 and CD56 expression

| *Culture* | *Treatment* | *% CD25⁺ cells* | *Mean channel* | *% CD56⁺ cells* | *Mean channel* |
|---|---|---|---|---|---|
| Exp.1 | | | | | |
| | IL-2 | 14 | N.E. | 8 | N.E. |
| | IL-2 + IL-4 | 8 | N.E. | 9 | N.E. |
| Exp.2 | | | | | |
| | IL-2 | 21 | 217 | 24 | 160 |
| | IL-2 + IL-4 | 14 | 122 | 26 | 94 |
| Exp.3 | | | | | |
| | IL-2 | 17 | 198 | 18 | 250 |
| | IL-2 + IL-4 | 15 | ŀ47 | 18 | 148 |

PBMC were incbated with 50 U $ml^{-1}$ of rIL-2 with or without 50 U $ml^{-1}$ of rIL-4. Aliquots were harvested at the day 4 and then phenotypic analysis was performed. N.E., not examined.

**Table III** Effect of IL-1 on IL-4 mediated inhibition

| | | *IL-1 (U ml⁻¹)* | | | |
|---|---|---|---|---|---|
| *Culture* | *Treatment* | *0* | *10* | *100* | *1000* |
| Exp.1 | | | | | |
| | IL-2 | 32.9 ± 1.3 | 50.6 ± 2.3 | 57.2 ± 5.2 | 79.1 ± 4.6 |
| | IL-2 + IL-4 | 17.3 ± 2.1 | 20.8 ± 2.1 | 25.7 ± 2.2 | 37.5 ± 1.9 |
| Exp.2 | | | | | |
| | IL-2 | 96.2 ± 12.3 | 111.8 ± 9.6 | 176.2 ± 25.1 | 190.4 ± 24.7 |
| | IL-2 + IL-4 | 35.4 ± 9.6 | 41.7 ± 4.3 | 114.1 ± 21.8 | 128.2 ± 12.5 |

PBMC were incubated with 50 U $ml^{-1}$ of rIL-2 with or without 50 U $ml^{-1}$ of rIL-4 in the presence of various amount of rIL-1β. The values represent LU per $10^6$ cells ± s.d.

**Table IV** Time course study of IL-1 mediated abrogation of inhibition

| | | | *IL-1 added at (h)* | | | |
|---|---|---|---|---|---|---|
| | *Culture condition* | *No IL-1* | *0* | *24* | *48* | *72* |
| Exp.1 | | | | | | |
| | IL-2 | 122.4 ± 7.3 | 162.5 ± 2.4 | 210.7 ± 8.9 | 182.7 ± 8.2 | 165.6 ± 5.1 |
| | IL-2 + IL-4 | 13.4 ± 2.0 | 72.6 ± 7.3 | 90.0 ± 9.8 | 43.2 ± 7.0 | 28.0 ± 5.3 |
| Exp.2 | | | | | | |
| | IL-2 | 40.2 ± 2.8 | 55.3 ± 1.7 | 57.0 ± 0.8 | 54.5 ± 1.9 | 51.3 ± 3.5 |
| | IL-2 + IL-4 | 12.1 ± 4.3 | 27.1 ± 2.2 | 22.8 ± 1.33 | 18.1 ± 3.4 | 16.6 ± 3.9 |

100 U $ml^{-1}$ of rIL-β was serially added to replicate 4 day cultures of PBMC and IL-2 (50 U $ml^{-1}$) with or without rIL-4 (50 U $ml^{-1}$). The values represent LU per $10^6$ cells ± s.d.

**Table V** Effect of IL1 on the expression of CD25 and CD56

| | | *% positive cells* | |
|---|---|---|---|
| | *Culture condition* | *CD25* | *CD56* |
| Exp.1 | | | |
| | Medium only | 6 | 9 |
| | IL-2 | 18 | 16 |
| | IL-2 + IL-4 | 11 | 15 |
| | IL-2 + IL-1 | 18 | 16 |
| | IL-2 + IL-4 + IL-1 | 14 | 14 |
| Exp.2 | | | |
| | IL-2 | 16 | 21 |
| | IL-2 + IL-4 | 11 | 21 |
| | IL-2 + IL-1 | 16 | 22 |
| | IL-2 + IL-4 + IL-1 | 13 | 19 |

PBMC were incubated for 4 days in the medium containing various mixtures of lymphokines indicated above, then aliquots were harvested and phenotypic analyses were performed. The final concentration of each cytokine used in the cultures was 50 U $ml^{-1}$.

PBMC incubated with IL-2 stained with anti-CD56 antibody at a peak channel at 27. While IL-1 alone increases (peak channel at 40) and IL-4 reduces (peak channel at 24) the intensity of CD56 expression, IL-1 does not abrogate the IL-4 mediated suppression of CD56 intensity (peak channel at 21).

We have also tested the effect of IL-4 on IL-2 induced IL-1 production. PBMC were cultured in IL-2 for 4 days and the cells were tested for LAK activity while their culture supernatants were tested for IL-1-beta. Results from Table VI show that IL-4 not only suppresses LAK cytotoxicity but also significantly decreases IL-2 induced IL-1 production.

**Table VI** Effect of IL-4 on IL-2 induced LAK activity and IL-1 production

| *Treatment (u ml⁻¹)* | *LAK cytotoxicity (LU ± s.d.)* | *IL-1 production (pg ml⁻¹)* |
|---|---|---|
| Control | 0 | 193 ± 20 |
| IL-2 (100) | 252 ± 57 | 690 ± 22 |
| IL-2 (100) + IL-4 (50) | 102 ± 8 | 123 ± 8 |
| IL-2 (1000) | 258 ± 58 | > 1000 |
| IL-2 (1000) + IL-4 (50) | 117 ± 16 | 389 ± 18 |

PBMC were cultured with indicated cytokines for 4 days; the cultured cells were tested for LAK cytotoxicity against M14 and the culture supernatants were tested for IL-1 concentration by EIA.

### Discussion

IL-4 inhibits the IL-2 induced LAK development of human PBMC (Widmer *et al.*, 1987; Spits *et al.*, 1988). Our data not only support these previous reports, but also show that suppression of LAK is dependent on the dose of IL-4 (Table I), and the suppression is effective over wide range of IL-2 concentration (10–1,000 U $ml^{-1}$) (Figure 1).

IL-4 added more than 24 h after the initiation of IL-2 culture does not suppress LAK induction (Figure 2). Similarly, other investigators have reported that IL-4 suppresses LAK development only at the initiation of the IL-2 culture (Spits *et al.*, 1988). IL-4 has also been reported to inhibit the activation of NK cells induced by short-term (18 h) incubation with IL-2 without any involvement of regulatory cells (Nagler *et al.*, 1988). This is in marked contrast to the effect of IL-4 in enhancing MLC induced CTL induction or influenza virus specific CTL induction. These effects are more profound if IL-4 is added at the later phase of incubation (Horohov et al., 1988; Widmer et al., 1987). The differences between CTL and LAK not only suggest that IL-4 has different effects on different cytotoxic cells, but also indicate that the effects of IL-4 depend on the different developmental stage of the cytotoxic cells. With NK/LAK cells, IL-4 appears to act on an early phase in development of augmented cytotoxicity.

One candidate for an early event sensitive to IL-4 suppression would be the induction of IL-2 receptors (CD25). Therefore, we tested the effects of IL-4 on CD25 and CD56 expression. IL-4 suppresses the IL-2 induced expression of CD25 but not CD56 (Table II). These data confirmed the report of Brooks and Rees (1988) that IL-4 suppresses the IL-2 induced expression of CD25 in both percentage of positive cells and intensity of staining. In contrast, our results indicate that the IL-4 does not suppress the percentage of IL-2 induced CD56 expressing cells, but does reduce the intensity of CD56 expression. This suggests that inhibition of CD56 or CD25 expression may be a mechanism by which IL-4 inhibits LAK activity.

An alternative early event that might be affected by IL-4 might be IL-1 production. Recently, it has been shown that IL-4 inhibits the secretion of interleukin 1 (IL-1) from macrophages (Essner et al., 1989). This suggests that IL-4 mediated inhibition on LAK induction may be through the suppression of IL-1 production triggered by IL-2. As shown in the Tables III–V, IL-1 and IL-4 have antagonistic effects on some IL-2 induced PBMC responses. IL-1 promotes LAK development in a dose-dependent manner, while IL-4 suppresses this function (Table III). Synergism in LAK development by IL-1 is most effective 2 days after initiation of the culture and IL-4 is most suppressive when present from the beginning of the culture (Table IV). In other words, IL-4 is least suppressive at the point when IL-1 shows its most significant enhancement of LAK development. More importantly our data directly show that IL-4 can suppress IL-2 induced IL-1 production in PBMC (Table VI). These results strongly suggest that IL-4 mediated suppression of IL-1 production may contribute to the inhibition of LAK induction.

We postulated that CD25 modulates the late phase of LAK development but is not involved in the early phase of LAK induction (Shau & Golub, 1985). Differences in timing might explain the apparently divergent results with anti-CD25 antibodies which have been found to have either no effect on LAK induction (Tsudo et al., 1987) or to cause a partial blocking of LAK induction (Grimm et al., 1983; Shau et al., 1988). While the inhibition of LAK cytotoxicity by anti-CD25 is often marginal and much less pronounced than the inhibition of IL-2 induced lymphocyte proliferation (Siegel et al., 1987), the combination of anti-CD25 and antibodies specific for IL-2R-beta chain caused much greater inhibition of LAK than the latter alone (Phillips et al., 1989). This evidence suggests that CD25 does have some importance in the generation of LAK function.

IL-4 suppresses CD25 expression but the suppression can be partially reversed by IL-1. This result, combined with the fact that IL-1 can reciprocally regulate LAK function with IL-4, indicates that suppression of CD25 expression and/or reduced IL-1 production are likely to be involved in IL-4 suppression of LAK development. Neither cytokine alone or in combination changes the percentage of $CD56^+$ cells. However, IL-4 reduces and IL-1 enhances the intensity of CD56 expression individually. While IL-1 cannot reverse the IL-4 mediated suppression of CD56 intensity, it can partially reverse the suppression of cytotoxicity. Therefore, these data suggest that the expression of CD56 does not directly correlate the development of LAK activity. Our results in this study clearly indicate that IL-2 induced PBMC responses are subject to regulation by other cytokines. IL-1 and IL-4 have reciprocal effects of LAK development by IL-2, with IL-1 augmenting and IL-4 suppressing cytotoxicity.

We acknowledge the generous provision of IL-2 by Amgen, and IL-1 and IL-4 by Immunex. We also thank Mr Anthony Kim for excellent technical assistance. This work was supported in part by USPHS Grant CA 34442. Nobuo Ebina is a UCLA visiting scientist from the Department of Surgery, School of Medicine, Tohoku University, Sendai, Japan. David Gallardo is a recipient of Howard Hughes Medical Institute Fellowship.

### References

BROOKS, B. & REES, R.C. (1988). Human recombinant IL-4 suppresses the induction of human IL-2 induced lymphokine-activated killer (LAK) activity. *Clin. Exp. Immunol.*, **74**, 162.

BROWN, M., HU-LI, J. & PAUL, W.E. (1988). IL4/B cell stimulatory factor 1 stimulates T cell growth by an IL-2-independent mechanism. *J. Immunol.*, **141**, 50.

CRUMP, W.L. III, OWEN-SCHAUB, L.B. & GRIMM, E.A. (1989). Synergy of human recombinant interleukin 1 with interleukin 2 in the generation of lymphokine-activated killer cells. *Cancer Res.*, **49**, 149.

ESSNER, R., ECONOMOU, J.S., RHODES, K. MCBRIDE, W.H. & MORTON, D.L. (1989). IL-4 down-regulates IL-1 and TNF gene expression in human monocytes. *J. Immunol.*, **142**, 3857.

GAUSE, W.C., TAKASHI, T., MOUNTZ, J.D., FINKELMAN, F.D. & STEINBERG, A.D. (1988). Activation of $CD4^-$, $CD8^-$ thymocytes with IL4 vs IL1 + IL2. *J. Immunol.*, **141**, 2240.

GRIMM, E.A., ROBB, R.J., ROTH, J.A. & 4 others (1983). Lymphokine-activated killer cell phenomenon. III. Evidence that IL-2 is sufficient for direct activation of peripheral blood lymphocytes into lymphokine-activated killer cells. *J. Exp. Med.*, **158**, 1356.

HOROHOV, D.W., CRIM, J.A., SMITH, P.L. & SIEGEL, J.P. (1988). IL-4 (B cell-stimulatory factor 1) regulates multiple aspects of influenza virus-specific cell-mediated immunity. *J. Immunol.*, **141**, 4217.

KAWAKAMI, Y., ROSENBERG, S.A. & LOTZE, M. (1988). Interleukin 4 promotes the growth of tumor-infiltrating lymphocytes cytotoxic for human autologous melanoma. *J. Exp. Med.*, **168**, 2183.

KERN, D.E., PEACE, D.J., KLARNET, J.P., CHEEVER, M.A. & GREENBERG, P.D. (1988). IL-4 is an endogenous T cell growth factor during the immune response to a syngenic retrovirus-induced tumor. *J. Immunol.*, **141**, 2824.

MULE, J.J., SMITH, C.A. & ROSENBERG, S.A. (1987). Interleukin 4 (B cell stimulatory factor 1) can mediate the induction of lymphokine-activated killer cell activity directed against fresh tumor cells. *J. Exp. Med.*, **166**, 792.

MULE, J.J., KROSNICK, J.A. & ROSENBERG, S.A. (1989). IL-4 regulation of murine lymphokine-activated killer activity in vitro: effects on the IL-2-induced expansion, cytotoxicity, and phenotype of lymphokine-activated killer effects. *J. Immunol.*, **142**, 726.

NAGLER, A., LANIER, L.L. & PHILLIPS, J.H. (1988). The effect of IL-4 on human natural killer cells: a potent regulator of IL-2 activation and proliferation. *J. Immunol.*, **141**, 2349.

PEACE, H.F., KERN, D.E., SCHULTZ, K.R., GREENBERG, P.D. & CHEEVER, M.A. (1988). IL-4-induced lymphokine-activated killer cells: lytic activity is mediated by phenotypically distinct natural killer-like and T cell-like large granular lymphocytes. *J. Immunol.*, **140**, 3679.

PHILLIPS, J.H., TAKESHITA, T., SUGAMURA, K. & LANIER, L.L. (1989). Activation of natural killer cells via the p75 interleukin 2 receptor. *J. Exp. Med.*, **170**, 291.

PROSS, H.F., BAINES, M.G., RUBIN, P., SHRAGGE, P. & PATTERSON, M.S. (1981). Spontaneous human lymphocyte-mediated cytotoxicity against tulor target cells. IX. The quantitation of natural killer cell activity. *J. Clin. Immunol.*, **1**, 51.

RAMSDELL, F.J., SHAU, H. & GOLUB, S.H. (1988). Role of proliferation in LAK cell development. *Cancer Immunol. Immunother.*, **26**, 139.

SHAU, H. & GOLUB, S.H. (1985). Signals for activation of NK and NK-like activity. *Nat. Immun. Cell Growth Regul.*, **4**, 113.

SHAU, H., GRAY, J.D. & GOLUB, S.H. (1988). Studies on cytotoxicity generated in human mixed lymphocyte culture IV. Interleukin 2 alone or from mixed lymphocyte culture yields natural killer-like cytotoxic cells distinct from allospecific T lymphocytes. *Cancer Immunol. Immunother.*, **27**, 255.

SIEGEL, J.P., SHARON, M., SMITH, P.L. & LEONARD, W.J. (1987). The IL-2 receptor chain (p70): Role in mediating signals for LAK, NK, and proliferative activities. *Science*, **238**, 75.

SPITS, H., YSSEL, H., PALIARD, X., KASTELEIN, R., FIGDOR, C. & DE VRIES, J.E. (1988). IL-4 inhibits IL-2 mediated induction of human lymphokine-activated killer cells, but not the generation of antigen-specific cytotoxic T lymphocytes in mixed leukocyte cultures. *J. Immunol.*, **141**, 29.

TSUDO, M., GOLDMAN, C.K., BONGIOVANNI, K.F. & 5 others (1987). The p75 peptide is the receptor for interleukin 2 expressed on large granular lymphocytes and is responsible for the interleukin 2 activation of these cells. *Proc. Natl Acad. Sci. USA*, **84**, 5394.

WIDMER, M.B., ACRES, R.B., SASSENFELD, H.M. & GRABSTEIN, K.H. (1987). Regulation of cytotoxic cell populations from human peripheral blood by B cell stimulatory factor 1 (interleukin 4). *J. Exp. Med.*, **166**, 1447.